# The Relationship among Anxiety, Worry, Perceived Stress, Defense Mechanisms, and High Levels of Post-Traumatic Stress Symptoms: A Discriminant Analytic Approach

**DOI:** 10.3390/jpm13020237

**Published:** 2023-01-28

**Authors:** Alessio Gori, Eleonora Topino, Alessandro Musetti

**Affiliations:** 1 Department of Health Sciences, University of Florence, Via di San Salvi 12, Pad. 26, 50135 Firenze, Italy; 2 Integrated Psychodynamic Psychotherapy Institute (IPPI), Via Ricasoli 32, 50122 Florence, Italy; 3 Department of Human Sciences, LUMSA University of Rome, Via della Traspontina 21, 00193 Rome, Italy; 4 Department of Humanities, Social Sciences and Cultural Industries, University of Parma, Borgo Carissimi 10, 43121 Parma, Italy

**Keywords:** PTSD, post-traumatic stress symptoms, impact of event, discriminant analysis, MANOVA, anxiety, perceived stress, worry, defense style

## Abstract

Post-traumatic stress disorder (PTSD) is a pathological condition that may lead to a significant deterioration in the quality of life over time. Therefore, the study of the elements that can characterize the disorder could be considered of great clinical interest and relevance. The aim of the present research was to empirically discriminate the influence of perceived stress, state anxiety, worry, and defense mechanisms (mature, neurotic, and immature) at different levels of post-traumatic stress symptoms. A sample of 1250 participants (69.5% women, 30.5% men; *M_age_* = 34.52, *SD* = 11.857) completed an online survey including the Impact of Event Scale—Revised, Ten-Item Perceived Stress Scale, Penn State Worry Questionnaire, Forty Item Defense Style Questionnaire, and State-Trait Anxiety Inventory—Form X3. Data were analysed by implementing MANOVA and discriminant analysis. Results showed significant differences in the levels of perceived stress, state anxiety, and worry, as well as neurotic and immature defenses based on the levels of post-traumatic stress symptoms: *F*(12,2484) = 85.682, *p* < 0.001; Wilk’s Λ = 0.430. Furthermore, these variables discriminate significant accuracy between participants who reported a mild psychological impact and those with a probable presence of PTSD, with perceived stress, which was found to be the best predictor. Classification results indicated that the original grouped cases were classified with 86.3% overall accuracy. Such findings may provide useful insight for clinical practice.

## 1. Introduction

Post-traumatic stress disorder (PTSD) is a widespread pathological condition that occurs following exposure to potentially traumatic events perceived as outside normal human experiences [1]. This is a common and complex mental disorder, described in the latest version of the *Diagnostic and Statistical Manual of Mental Disorders* (DSM-5-TR) [2] with four clusters of symptoms that can be identified as intrusion (i.e., re-experiencing traumatic events through flashbacks, nightmares, and intrusive memories), avoidance (i.e., avoidance of the stimuli related to the traumatic events and suppression of re-experiencing phenomena), negative alterations of cognition and mood (i.e., worsening mood and cognition processes after the traumatic events), and hyperarousal (i.e., marked alterations in arousal and reactivity), such as to impair the individual’s functioning in important areas of his life and cause clinically significant distress. Indeed, post-traumatic stress disorder can have long-term harmful effects on an individual’s social dimension, family life, and personal health [3]. It has been associated with worse physical health [4] and considerable medical comorbidities, such as chronic inflammation, cardiometabolic disorders, and increased risk of dementia (see Lohr et al., [5]; Rosenbaum et al., [6]; Yehuda et al., [7], for literature reviews). Furthermore, post-traumatic stress symptoms have also been related to mental illness, as highlighted by previous research showing associations with suicidal ideation [8], depression [9], hostility [10], lower levels of self-efficacy [11], and a lower perception of life satisfaction [12,13], to name a few.

Given this evidence and since post-traumatic stress disorder can cause loss of personal, social, and material resources, leading to a significant deterioration in the quality of life over time [14,15], the study of the elements that can characterize the disorder, contributing to post-traumatic stress symptom severity and/or to the resulting consequences, appears to be of great clinical interest and relevance. In this line, the present research explored the discriminant role of perceived stress, state anxiety, worry, and defense mechanisms (mature, neurotic, and immature) at different levels of post-traumatic stress symptoms. Following is a brief review of research evidence showing their association with post-traumatic stress disorder.

### 1.1. Post-Traumatic Stress Disorder and Stress

A dimension inherent in this condition can be identified in stress, defined as a complex psychophysiological state that includes a series of response processes to a real or perceived threat to homeostasis [16]. High levels of perceived stress can lead to physical problems, such as chronic pain [17], cardiovascular diseases [18], and weakened immune responses [19], and it may negatively affect psychological well-being, showing significant relationships with aspects such as depression [20], substance use [21], and emotional exhaustion [22]. Consistently, different evidence has underlined the relation between this variable and post-traumatic stress disorder, highlighting that higher levels of perceived stress are associated with greater severity of post-traumatic stress symptoms [23,24].

### 1.2. Post-Traumatic Stress Disorder and Anxiety

State anxiety can be another element linked to post-traumatic stress disorder [25]. Anxiety may be defined as a negative emotional state, characterized by tension, discomfort, and apprehension about the possibility of physical or psychological damage, often accompanied by physiological activation [26,27]. When this occurs at extreme and disproportionate levels to the stimulus, it becomes pathological and can interfere with daily functioning [28], presenting negative correlations with subjective well-being [29,30] and satisfaction with life [13]. Previous evidence shows that anxiety is a risk factor for post-traumatic stress disorder severity [31], and it may have an exacerbating and maintenance role [32], feeding a state of awareness of threat and danger [33].

### 1.3. Post-Traumatic Stress Disorder and Worry

A further variable that can discriminate between different post-traumatic stress disorder severity profiles is worry, i.e., a form of repetitive negative thinking [34] characterized by engagement in mental problem solving concerning issues whose outcome is uncertain and includes one or more negative possibilities [35]. Excessive and persistent levels of worry can relate to worse mental health [36], dysfunctional responses to stress [37], and greater severity of symptoms of post-traumatic stress disorder following different types of traumatic events [38,39,40]. Indeed, although in the short term this variable may be associated with reductions in physiological and/or emotional arousal in individuals with post-traumatic stress disorder, a chronic and persistent tendency to worry can interfere with adaptive cognitive and emotional processing, contributing to the maintenance or worsening of post-traumatic stress symptoms [41].

### 1.4. Post-Traumatic Stress Disorder and Defense Mechanisms

Finally, a consolidated line of research highlights the role of defense mechanisms concerning psychopathology [42,43], including post-traumatic stress disorder [44,45,46]. Defense mechanisms are mental operations, generally unconscious and automatic, to protect the self from internal conflicts or stressful situations [47,48] and can be classified on a continuum of increasing cognitive distortion, which starts from mature styles characterized by absent or limited cognitive distortion, passes through mature styles, and finds immature styles at the other extreme [49,50]. While mature defenses have been highlighted as protective factors for mental health [51], the excessive use of immature and neurotic defense mechanisms was associated with harmful effects [52], including post-traumatic stress symptoms [45,53,54].

### 1.5. Aim and Hypotheses

Based on the aforementioned literature, the core aim of the present study was to empirically discriminate the influence of perceived stress, state anxiety, worry, and defense mechanisms (mature, neurotic, and immature) at different levels of post-traumatic stress symptoms. 

Therefore, the research was developed with the goal of exploring whether groups with different levels of post-traumatic stress symptoms (normal scores, mild psychological impact, and probable presence of PTSD) show differences in the observed variables and whether these differences are relevant to characterize and discriminate the most clinically relevant condition (probable presence of PTSD) from medium risk conditions (mild psychological impact).

The first hypothesis was that significant differences in the variables based on the levels of post-traumatic stress symptoms will be found, such as high scores of perceived stress, state anxiety, and worry, as well as a dysfunctional use of defense mechanisms were greater as the risk of PTSD increased (**H1**).

Additionally, it was expected that the variables showing significant differences will allow for effective discrimination of the most clinically relevant condition (probable presence of PTSD) from medium risk conditions (mild psychological impact; **H2**).

## 2. Materials and Methods

### 2.1. Participants and Procedures

A sample of 1250 participants (69.5% women, 30.5% men; *M_age_* = 34.52, *SD* =11.857) was involved in this research. Using snowball sampling, they were recruited online by sending out a link to an online survey hosted on the Google Forms platform. Participation was voluntary, and no payment was offered for involvement in the study. Before starting the survey, all participants were informed about the general aim of the research and that their answers would be used for research purposes in an aggregated and anonymous way. Furthermore, they provided informed consent electronically. All procedures were approved by the Ethical Committee of the Integrated Psychodynamic Psychotherapy Institute (IPPI).

### 2.2. Measures

#### 2.2.1. Impact of Event Scale—Revised (IES-R)

The *Impact of Event Scale—Revised* (IES-R; Weiss & Marmar [55]; Italian version: Craparo et al., [56]) is a 22-item self-reporting measure for the assessment of post-traumatic symptoms. Items (e.g., “*I felt watchful and on-guard*”) are scored on a five-point Likert scale, from 0 (not at all) to 4 (extremely), and may be grouped into the factors of intrusion, avoidance, and hyperarousal. A total IES-R score of 33 or higher has been considered the best cut-off to indicate a probable presence of PTSD [57], while scores between 24 and 32 indicate a mild psychological impact and PTSS, and a score equal to or lower than 23 can be considered normal [58]. In this study, the total score of the Italian version was used and showed excellent internal consistency in the present sample (*α* = 0.92).

#### 2.2.2. Ten-Item Perceived Stress Scale (PSS−10)

The *Ten-Item Perceived Stress Scale* (PSS−10; Cohen, & Williamson [59]; Italian version: Fossati [60]) is a 10-item self-reporting measure for the assessment of the level of stress experienced by the respondents. Items (e.g., “*In the last month, how often have you found that you could not cope with all the things that you had to do?*”) are scored on a five-point Likert scale, from 0 (never) to 4 (very often). The Italian translation used in this study showed good internal consistency in the present sample (*α* = 0.88).

#### 2.2.3. Penn State Worry Questionnaire (PSWQ)

The *Penn State Worry Questionnaire* (PSWQ; Meyer, Miller, Metzger, & Borkove [61]; Italian version: Meloni & Grana [62]) is a 16-item self-reporting measure for the assessment of pervasive worry. Items (e.g., “*I know I shouldn’t worry about things, but I just cannot help it*”) are scored on a five-point Likert scale, from 1 (not at all typical) to 5 (very typical). The Italian version used in this study showed good internal consistency in the present sample (*α* = 0.87).

#### 2.2.4. Forty Item Defense Style Questionnaire (DSQ—40)

The *Forty Item Defense Style Questionnaire* (DSQ-40; Andrews et al., [49]; Italian version: Farma & Cortinovis [63]) is a 40-item self-reporting measure for the assessment of defense mechanisms. Items (e.g., “*I get satisfaction from helping others and if this were taken away from me I would get depressed*”) are scored on a nine-point Likert scale, from 1 (strongly disagree) to 9 (strongly agree) and are grouped into three styles: mature, neurotic, and immature. The Italian version used in this study showed an acceptable internal consistency in the present sample (mature, *α* = 0.60; neurotic, *α* = 0.61; immature, *α* = 0.82).

#### 2.2.5. State-Trait Anxiety Inventory—Form X3 (STAI—X3)

The *State-Trait Anxiety Inventory—Form X3* (STAI—X3; Spielberger, Gorsuch, & Lushene [64]; Italian version: Vidotto & Bertolotti [65]) is a 10-item self-reporting measure for the assessment of state anxiety. Items (e.g., “*I feel nervous*”) are scored on a five-point Likert scale, from 1 (not at all) to 4 (very much so). The Italian version used in this study showed good internal consistency in the present sample (*α* = 0.94).

### 2.3. Analytic Plan

Data analyses were performed by using the SPSS statistical software (v. 21.0, IBM, Armonk, NY, USA). Frequencies based on IES-R values were calculated. Differences in the levels of perceived stress, worry, and defense mechanisms (mature, neurotic, and immature) based on the severity of post-traumatic symptoms (normal scores, mild psychological impact, and probable presence of PTSD) were assessed by implementing a multivariate analysis of variance (MANOVA). Follow-up tests with separate analyses of variance (ANOVAs) with a Bonferroni-adjusted *p*-value of 0.008 and post hoc analyses using a Scheffé test were implemented. Then, the subsample including only the mild psychological impact and probable presence of PTSD groups was randomly split. In line with the results of the previous analyses, discriminant function analysis was used in the first random 50% of this subsample to explore the relative contributions of perceived stress, state anxiety, and worry, as well as neurotic and immature defenses to differentiate the two higher levels of psychological impact groups (mild psychological impact and probable presence of PTSD). Discriminant loadings of more than 0.30 were considered meaningful [66]. Split-half cross-validation was then performed, by exploring a classification analysis on the second half of the subsample. Except for the ANOVAs, significance was set at *p* < 0.05.

## 3. Results

Based on the IES-R cut-off, three groups were identified: most of the respondents (N = 500; 40%) showed scores indicative of a probable presence of PTSD and 300 (24%) reported a mild psychological impact, while 450 (36%) participants showed scores that can be considered in the normal range.

The MANOVA highlighted a statistically significant difference in the observed variables based on the levels of post-traumatic distress symptoms: *F*(12, 2484) = 85.682, *p* < 0.001; Wilk’s Λ = 0.430. 

Specifically, the ANOVAs and the Scheffé test indicated that the group with normal scores showed significantly lower values than the one with mild psychological impact, which in turn reports significantly lower values than those with probable presence of PTSD concerning perceived stress (*F*_2, 1247_ = 604.722, *p* < 0.001), state anxiety (*F*_2, 1247_ = 468.769, *p* < 0.001), worry (*F*_2, 1247_ = 308.536, *p* < 0.001), neurotic defense mechanism (*F*_2,1247_ = 87.672, *p* < 0.001), and immature defense mechanism (*F*_2, 1247_ = 98.872, *p* < 0.001), while no significant differences were found in the use of mature defenses: *F*_2, 1247_ = 2.919, *p* = 0.054 (see Table 1).

Concerning discriminant analysis, the statistical function was found to be statistically significant, indicating that the predictor variables (perceived stress, state anxiety, and worry, as well as neurotic and immature defenses) differentiated across the two higher levels of psychological impact (mild psychological impact and probable presence of PTSD): Wilks’s Λ = 0.556, *χ2* (5) = 230.759, *p* < 0.001 (see Table 2).

All the discriminant loadings were above the cut-off of 0.30 (see Table 3), and they ranged from 0.309 (neurotic defenses) to 0.887 (perceived stress).

Classification results indicated that the original grouped cases were classified with 86.3% overall accuracy. Concerning the group of participants who showed a mild psychological impact, the classification accuracy was 71.6%, while for those with a probable presence of PTSD it was 94.9% (see Table 4). The cross-validated results supported the original accuracy levels with an overall accuracy of 85.6% reported.

## 4. Discussion

Post-traumatic stress disorder (PTSD) is a debilitating condition that weighs heavily on patients, their families, and society at large. Indeed, post-traumatic stress disorder appreciably affects the quality of professional, familial, and social areas [2,7] and may result in a significant and considerable deterioration in the quality of life [67,68], as estimated both in cross-sectional [69] and longitudinal [70,71] research. Given the clinical relevance of this condition, this study aimed to expand research on the mechanisms underlying the disorder, exploring the role of perceived stress, state anxiety, worry, and defense mechanisms (mature, neurotic, and immature) in effectively discriminating between different levels of post-traumatic stress symptom severity.

The results partially confirmed the first hypothesis (**H1**), since groups with different levels of post-traumatic stress symptoms (normal scores, mild psychological impact, and probable presence of PTSD) differ significantly for all variables, except for the mature defense style. Further research is needed to clarify this aspect. Indeed, previous studies highlighted the protective role of mature defenses against the impact of the event and post-traumatic symptoms [45]. However, it should also be highlighted that psychological health is not only linked to the application of mature defense strategies but above all to the appropriate and integrated use of a variety of defenses according to the circumstances [46]. Consistently, as the risk of PTSD increases, the neurotic and immature defenses also grow, as well as perceived stress, state anxiety, and worry, in line with previous evidence that highlighted significant and positive associations between these variables and the levels of post-traumatic stress symptoms [24,26,33,38,46,72,73,74].

These results are further detailed by the discriminant analysis, which added to the interpretation of the findings showing that perceived stress is the best predictor in discriminating between participants who reported mild psychological impact and those with a probable presence of PTSD. This echoes previous evidence that has identified higher levels of perceived stress in individuals with post-traumatic stress disorder than in controls [74] and is consistent with a line of research that highlights significant and positive associations between perceived stress and post-traumatic stress symptoms in different populations, such as civilian exposure to ongoing terrorist attacks [23], patients with oral cancer [24], or the population after the COVID-19 pandemic [75]. Overall, the whole equation, composed of perceived stress, state anxiety, and worry, as well as neurotic and immature defense mechanisms, allows for discrimination with significant accuracy between subjects with mild and those with higher risk (sensitivity = 73.3%; specificity = 93.6%), supporting the second hypothesis (**H2**). Although stress, anxiety, and worry are not pathological in themselves, their chronic manifestation appears consistent with the hyperactivation and threat alertness of post-traumatic stress disorder [32]. This may lead to a reduction in executive processing resources, which are functional for the effective emotional processing of a traumatic experience and necessary for more adaptive emotional control and coping [37]. Furthermore, individuals exposed to traumatic events are more likely to affectively distort environmental information by adopting dysfunctional defense mechanisms [76].

### Limitations and Suggestions for Future Research

This study presents some limitations that must be acknowledged. First, data were self-reported. This may be a source of reporting biases, a key problem in the assessment of most observational research study designs [77]. The use of a multimodal approach (e.g., integrating self-reporting measures and interviews) may be an interesting way to overcome this issue in future research. Moreover, post-traumatic stress disorder is a complex and articulated condition that can be associated with numerous factors [3,78,79], and involves a gene-environment interaction (see Mehta and Binder [80], Afifi and colleagues [81], Koenen, Nugent, and Amstadter [82] for reviews). Therefore, the results of this study can offer further knowledge of this phenomenon, without however being exhaustive. Future research is needed to integrate these data by also exploring the role of other elements, to further understand the variables characterizing post-traumatic stress disorder. Consistently, in this study, 40% of the participants showed scores indicative of a probable presence of PTSD, and this is in line with other research involving the Italian population during the years following the COVID-19 pandemic [83,84]. The exploration of the pandemic’s impact on mental health is an important field of study [85,86], and the replication of these results also integrating a detailed assessment of the influence of aspects related to COVID-19 on post-traumatic stress disorder can be an important goal for future research. Furthermore, study was based on an online sampling process, recruiting participants through a snowball technique. Although snowball sampling showed to be a viable method of recruiting study participants [87], the replication of the results by implementing. probability sampling procedures could be an important challenge for future research. Furthermore, no measures have been included in the assessment of the type of trauma. Although there is an open debate in the literature on whether or not this aspect is necessary [88,89], future research could replicate these results by integrating this assessment.

## 5. Conclusions

Given the negative impact in different areas of life and the functional deterioration resulting from post-traumatic stress disorder [12,90], research on the psychological variables associated with this disorder acquires particular relevance from a potential clinical and therapeutic application. The present study highlighted significant differences in the levels of perceived stress, state anxiety, and worry, as well as neurotic and immature defense mechanisms based on the risk for PTSD, also showing the discriminating capacity of these variables between participants who reported a mild psychological impact and those with a probable presence of PTSD. Such data may have interesting practical implications. Indeed, psychotherapy can be successful in limiting post-traumatic stress disorder and increasing the quality of life [91,92], and the results of this study may offer a better understanding of the specific contributions of some factors to post-traumatic symptoms, suggesting the usefulness of incorporating treatment elements aimed at these aspects. In this regard, the scientific literature highlighted the effectiveness of different treatment for worry or anxiety (e.g., see Hanrahan and colleagues [93], Normann and Morina [94], and Mayo-Wilson and colleagues [95] for meta-analyses), as well as for perceived stress in different samples (such as Patients with Irritable Bowel Syndrome [96] or pregnant women [97]). Furthermore, defense mechanisms have been the object of a large field psychotherapy theory, research, and practice, given the associations between the improvement in defensive functioning with change in symptoms and functioning (see Perry and Bond [98] for a review). Summing up, since the results of this research show that higher levels of perceived stress, state anxiety, worry, and neurotic and immature defenses were associated with higher severity of post-traumatic stress symptoms, these data underline the need to work therapeutically on these dimensions to increase the quality of life and the subjective well-being of subjects with a probable presence of PTSD.

## Figures and Tables

**Table 1:** Means, standard deviation and comparisons of perceived stress, state anxiety, worry, and defense mechanisms (mature, neurotic, and immature) based on the levels of post-traumatic stress symptoms.

	Normal Scores (N = 794)		Mild Psychological Impact (N = 415)		Probable Presence of PTSD (N = 781)		*F*	*p*	Bonferroni Post Hoc
	*M*	*SD*	*M*	SD	*M*	*SD*			
Perceived stress	13.567	5.920	17.264	6.121	25.823	4.804	604.722	**< 0.001**	G3 > G2 > G1
State anxiety	16.100	4.631	19.082	6.384	27.521	6.648	468.769	**<0.001**	G3 > G2 > G1
Worry	38.523	11.529	45.894	11.717	57.237	11.822	308.536	**<0.001**	G3 > G2 > G1
Mature defenses	43.651	9.115	43.377	8.184	42.286	9.663	2.919	0.054	-
Neurotic defenses	30.023	9.905	32.762	8.378	37.930	9.341	87.672	**<0.001**	G3 > G2 > G1
Immature defenses	85.122	24.083	90.030	23.401	106.457	25.051	98.872	**<0.001**	G3 > G2 > G1

***Note:*** Bold values indicate *p* within the criteria of significance (*p* < 0.008); G1 = normal scores; G2 = mild psychological impact; G3 = probable presence of PTSD.

**Table 2:** Summary of the discriminant function.

Function	Eigenvalue	% of Variance	Canonical Correlation	Wilks’ Lambda	Chi-Square	df	*p*
1	0.798	100.0	0.666	0.556	230.759	5	<0.001

**Table 3:** Structure coefficients (structural matrix).

Measured Variable	Coefficients
	1
Perceived stress	0.887
State anxiety	0.708
Worry	0.586
Immature defenses	0.356
Neurotic Defenses	0.309

**Table 4:** Classification results.

		Psychological Impact	Predicted Group Membership		Total
			Mild Psychological Impact	Probable Presence of a PTSD	
Original	Count	Mild psychological impact	106	42	148
		Probable presence of a PTSD	13	241	254
	%	Mild psychological impact	71.6	28.4	100.0
		Probable presence of a PTSD	5.1	94.9	100.0
Cross-validated ^b^	Count	Mild psychological impact	104	44	148
		Probable presence of a PTSD	14	240	254
	%	Mild psychological impact	70.3	29.7	100.0
		Probable presence of a PTSD	5.5	94.5	100.0

***Note:*** 86.3% of original grouped cases correctly classified. ^b^. Cross validation is performed only for those cases in the analysis. In cross validation, each case is classified by the functions derived from all cases other than that case. 85.6% of cross-validated grouped cases correctly classified.

## Data Availability

The data presented in this study are available on request from the corresponding author.
